# Supplementary material for: Mitochondrially-targeted APOBEC1 is a potent mtDNA mutator affecting mitochondrial function and organismal fitness in Drosophila
Source: Nat Commun. 2019 Jul 23;10:3280. doi: 10.1038/s41467-019-10857-y (PMC6650417; doi:10.1038/s41467-019-10857-y)
Supplement: Supplementary file 1 — Supplementary Information [file 41467_2019_10857_MOESM1_ESM.pdf]

Mitochondrially-targeted APOBEC1 is a potent mtDNA mutator affecting mitochondrial function and organismal fitness in *Drosophila*

Andreazza et al.

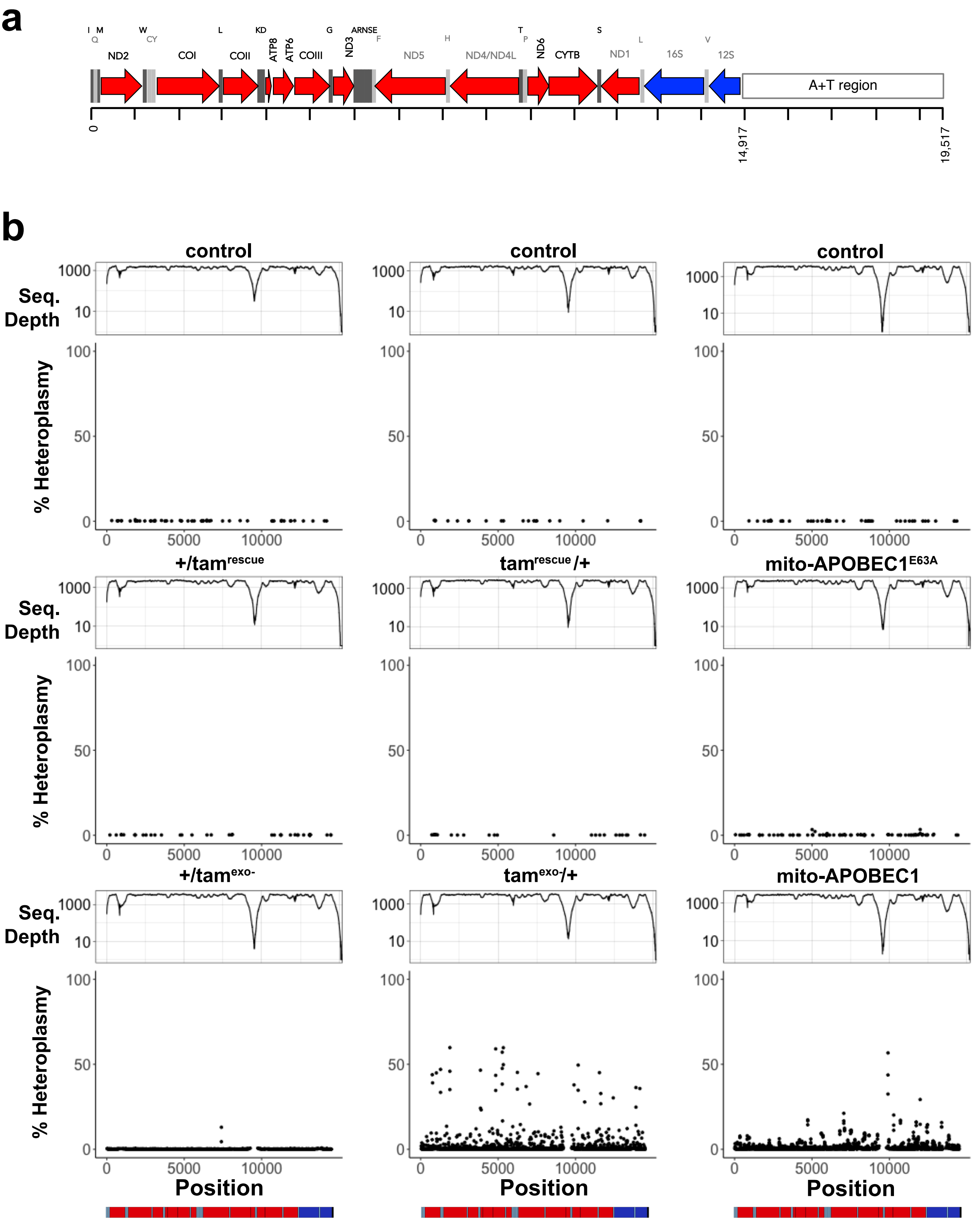

Supplementary Figure 1. Coverage of Duplex Sequencing across the mitochondrial genome and heteroplasmy levels recovered at each position.

(**a**) Detailed schematic of the *Drosophila* mtDNA genome map. Red: protein coding genes; grey: tRNAs; dark blue: rRNAs. Gene names and one-letter tRNA symbols are indicated. (**b**) Sequencing depth (log_{10}-transformed *y*-axis) and heteroplasmy level of mutations across the mitochondrial genome in 10-day-old flies of indicated genotypes. Each dot represents an identified mutation shown at the observed heteroplasmy level. Each chart shows the sum of all mutations identified per genotype (n=3 animals). Source data are provided as Supplementary Data 1.

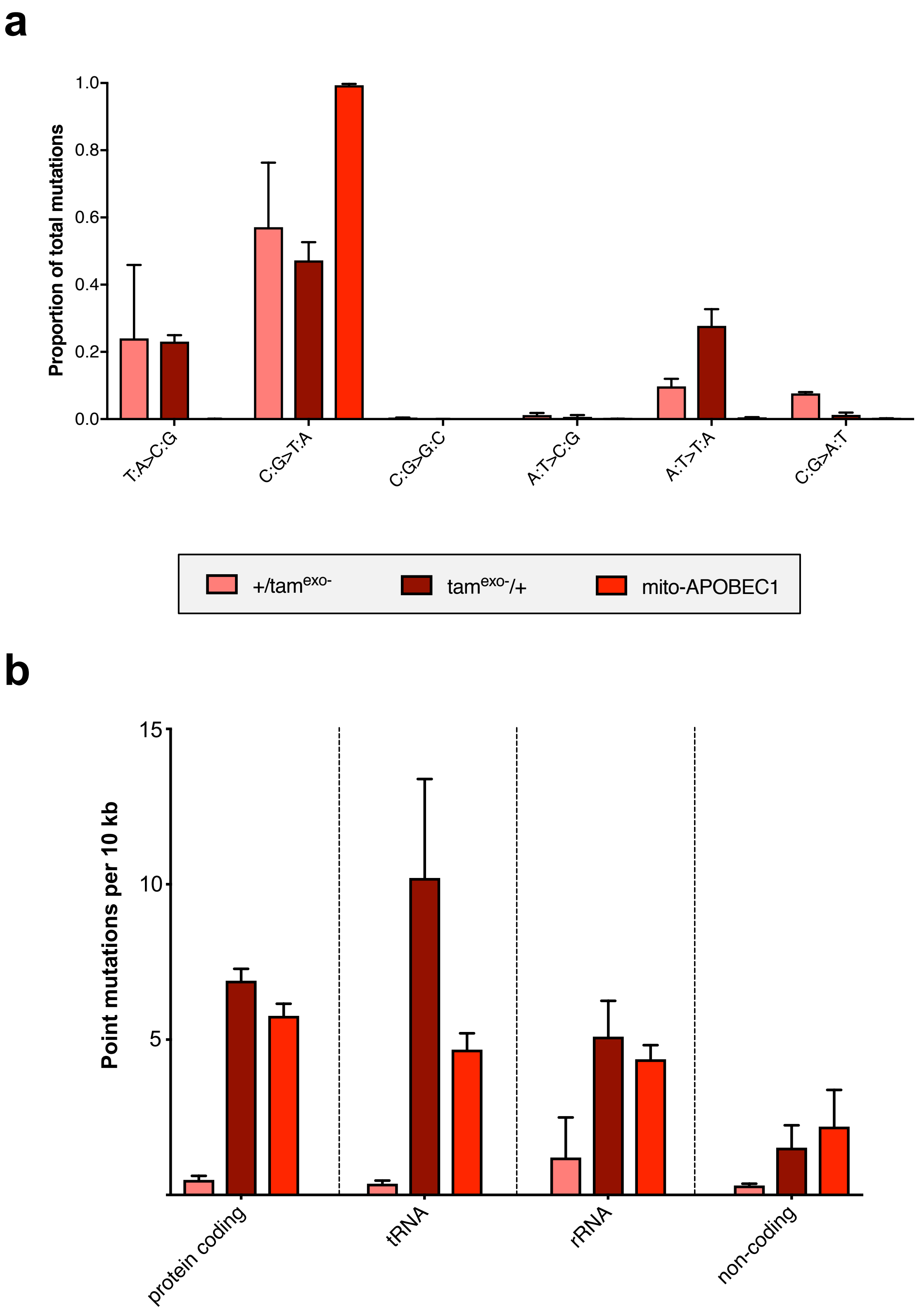

Supplementary Figure 2. *mito-APOBEC1* is highly specific for C:G>T:A transitions across the genome.

(**a**) Proportion of total mutations observed expressed by mutation type, as indicated; and (**b**) point mutation load in protein coding genes, tRNAs, rRNAs or non-coding sequence for paternally inherited *tam*$^{exo-}$ (+/*tam*$^{exo-}$), maternally inherited *tam*$^{exo-}$ (*tam*$^{exo-}$/+) and *mito-APOBEC1* mutator flies. Charts show mean ± SD, n=3 animals. Source data are provided as a Source Data file.

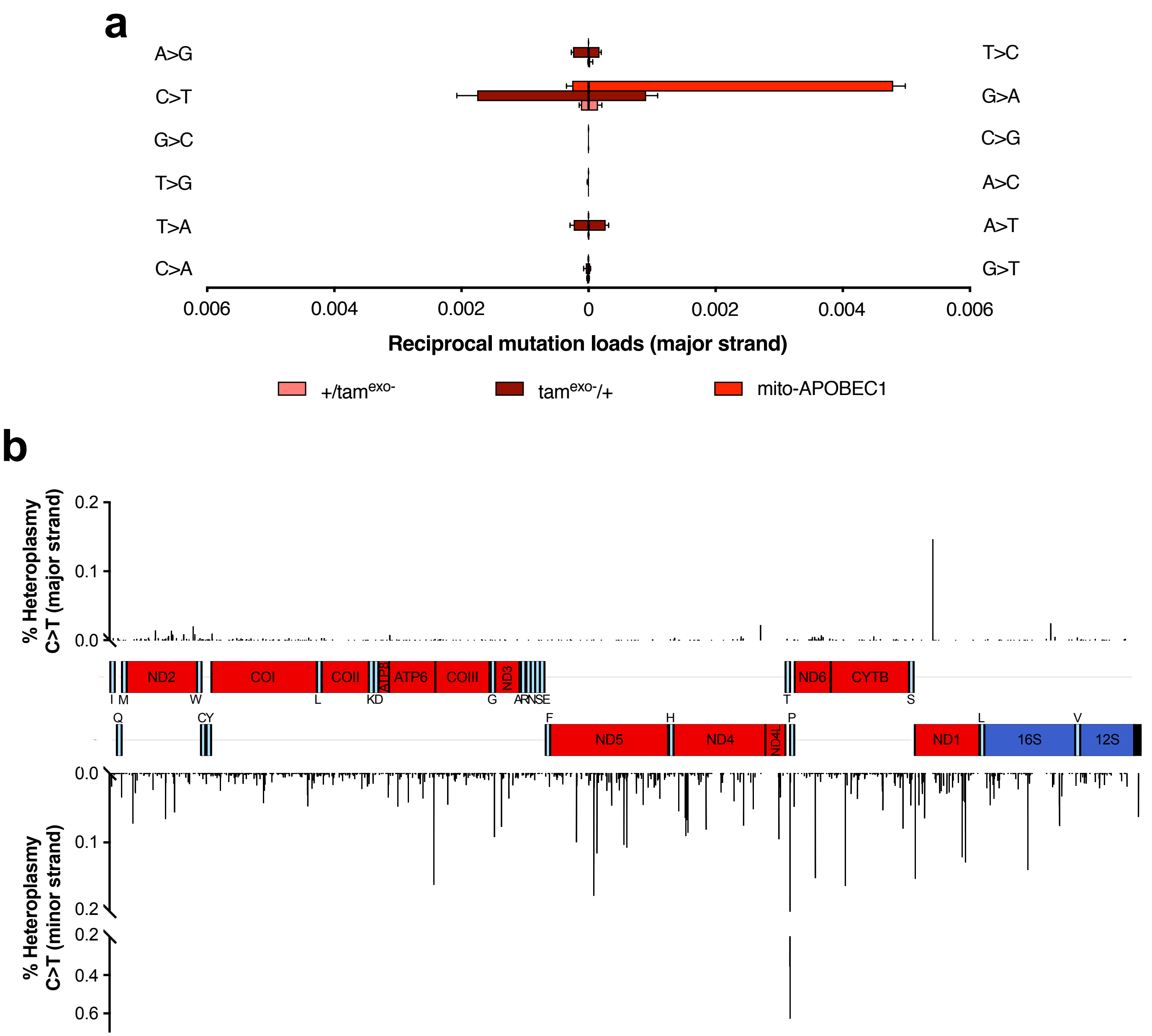

Supplementary Figure 3. *mito-APOBEC1* preferentially targets the minor strand.
(**a**) Frequency of reciprocal mutation types on the major strand (reference sequence) in 10-day-old paternally inherited *tam*$^{exo-}$ (+/*tam*$^{exo-}$), maternally inherited *tam*$^{exo-}$ (*tam*$^{exo-}$/+) and *mito-APOBEC1* mutator flies. Chart show mean ± SD, n=3 animals per genotype. (**b**) Distribution of average heteroplasmies for C>T mutations along the major strand (top) and minor strand (i.e. G>A substitutions in the major strand, bottom) in *mito-APOBEC1* flies*.* Genome map per each strand is depicted as red: protein coding genes; light blue: tRNAs; dark blue: rRNAs. Gene names and one-letter tRNA symbols are indicated. Source data are provided as a Source Data file and Supplementary Data 1.

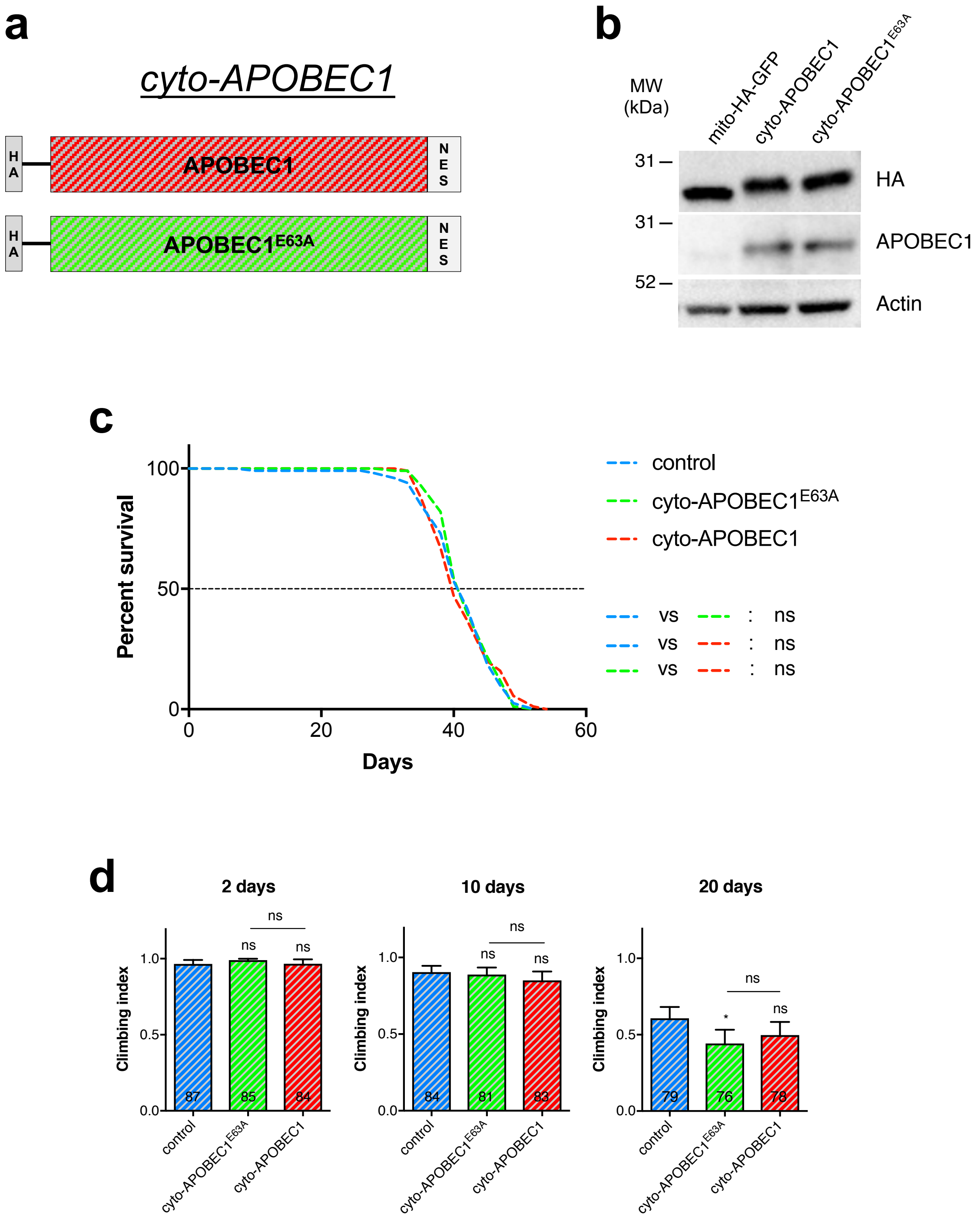

Supplementary Figure 4. Expression of *cyto-APOBEC1* does not cause lifespan or behavioural defects.

(**a**) Schematic of *cyto-APOBEC1* construct and inactive (E63A) mutant. (**b**) Immunoblot showing *cyto-APOBEC1* transgene expression in whole fly extracts, using both an anti-APOBEC1 antibody and anti-HA. Transgenic *mito-HA-GFP* expression is used as a positive control, and anti-Actin immunostaining as loading control. (**c**) Lifespan of *cyto-APOBEC1* flies is no different to that of *cyto-APOBEC1*^{E63A} and control flies expressing *mito-HA-GFP*. Log-rank (Mantel-Cox) test. (**d**) Climbing ability of *cyto-APOBEC1* flies is not affected across different ages. Kruskal-Wallis test with Dunn's multiple comparisons correction. The number of flies tested are shown per column. Charts show mean ± 95% confidence interval. * P<0.05. Source data are provided as a Source Data file.

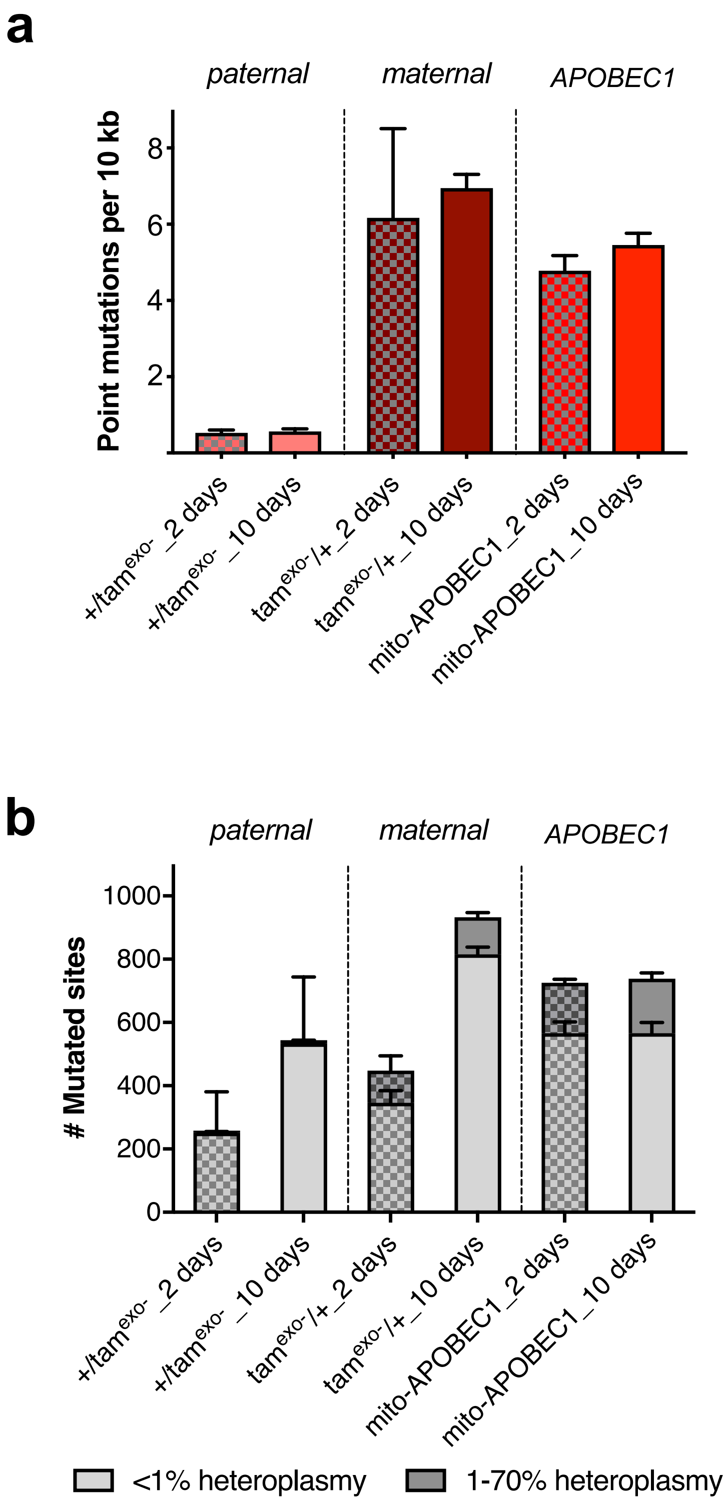

Supplementary Figure 5. The number of mutated sites increases in *tam*$^{exo-}$ but not in *mito-APOBEC1* flies over time.
(**a**) Point mutation load (**b**) and number of mutated sites in paternally inherited *tam*$^{exo-}$ (+/*tam*$^{exo-}$), maternally inherited *tam*$^{exo-}$ (*tam*$^{exo-}$/+) and *mito-APOBEC1* mutator flies at 2 and 10 days of age. Charts show mean ± SD, n=3 animals. Source data are provided as Source data file and Supplementary Data 1 and 3.

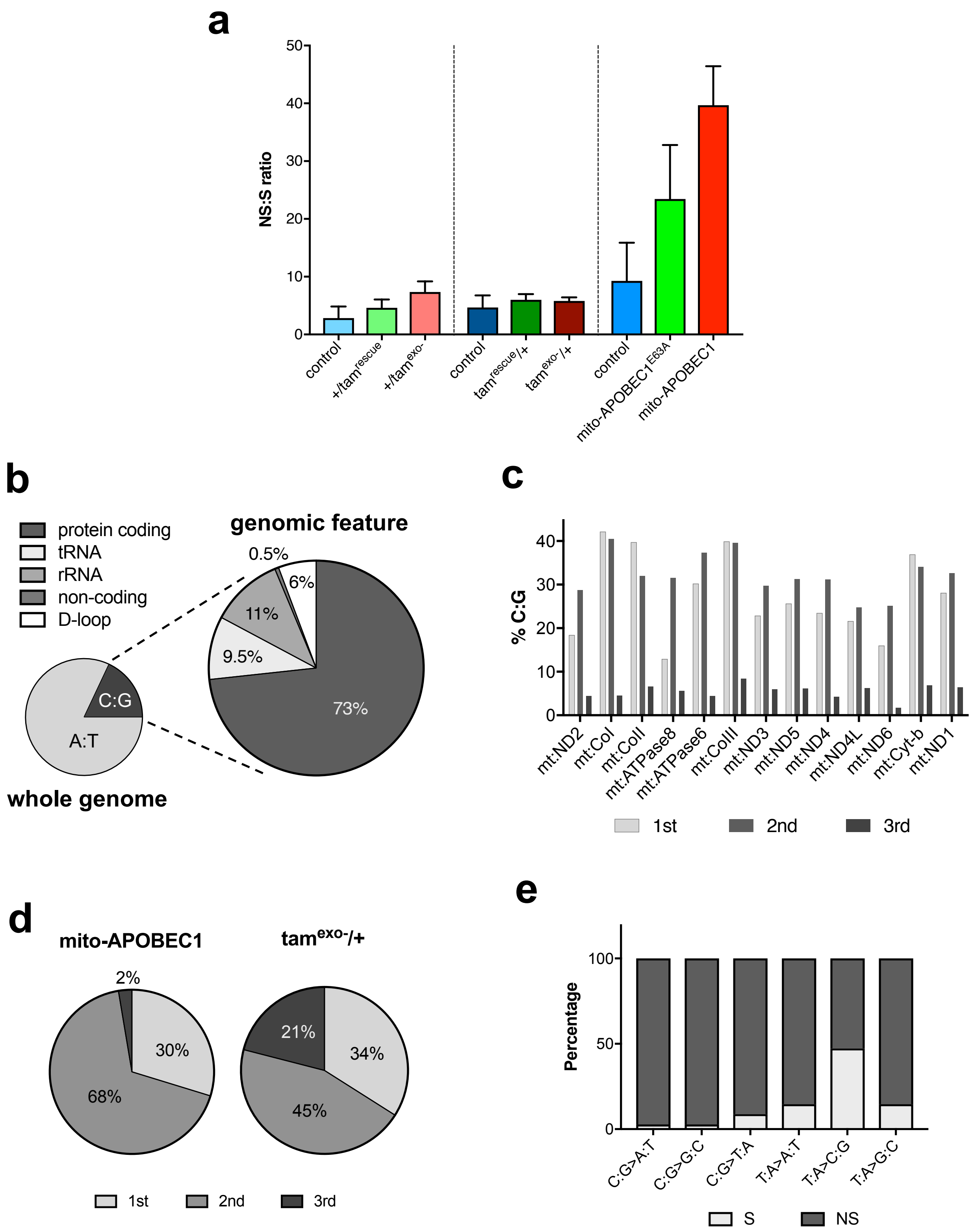

Supplementary Figure 6. *mito-APOBEC1* targets 1st and 2nd position in the codon, producing non-synonymous substitutions.

(**a**) Non-synonymous:synonymous (NS:S) ratio of all substitutions found in protein coding genes for mutator flies and respective controls. (**b**) Distribution of C:G nucleotides across the *Drosophila* mtDNA genome. C:G nucleotides represent 18% of the whole mtDNA genome; and most of them are located in protein coding sequences. (**c**) Percentage of C:G content per codon position for each of the mtDNA protein-coding genes. (**d**) Proportion of all protein-coding mutations from in *mito-APOBEC1* and maternally inherited *tam*$^{exo-}$ (*tam*$^{exo-}$/+) mutator flies in relation to their codon-base position. (**e**) Percentage of C:G and T:A mutations that produce either synonymous (S) or non-synonymous (NS) substitutions based on *Drosophila* mtDNA reference sequence. Source data are provided as a Source Data file.

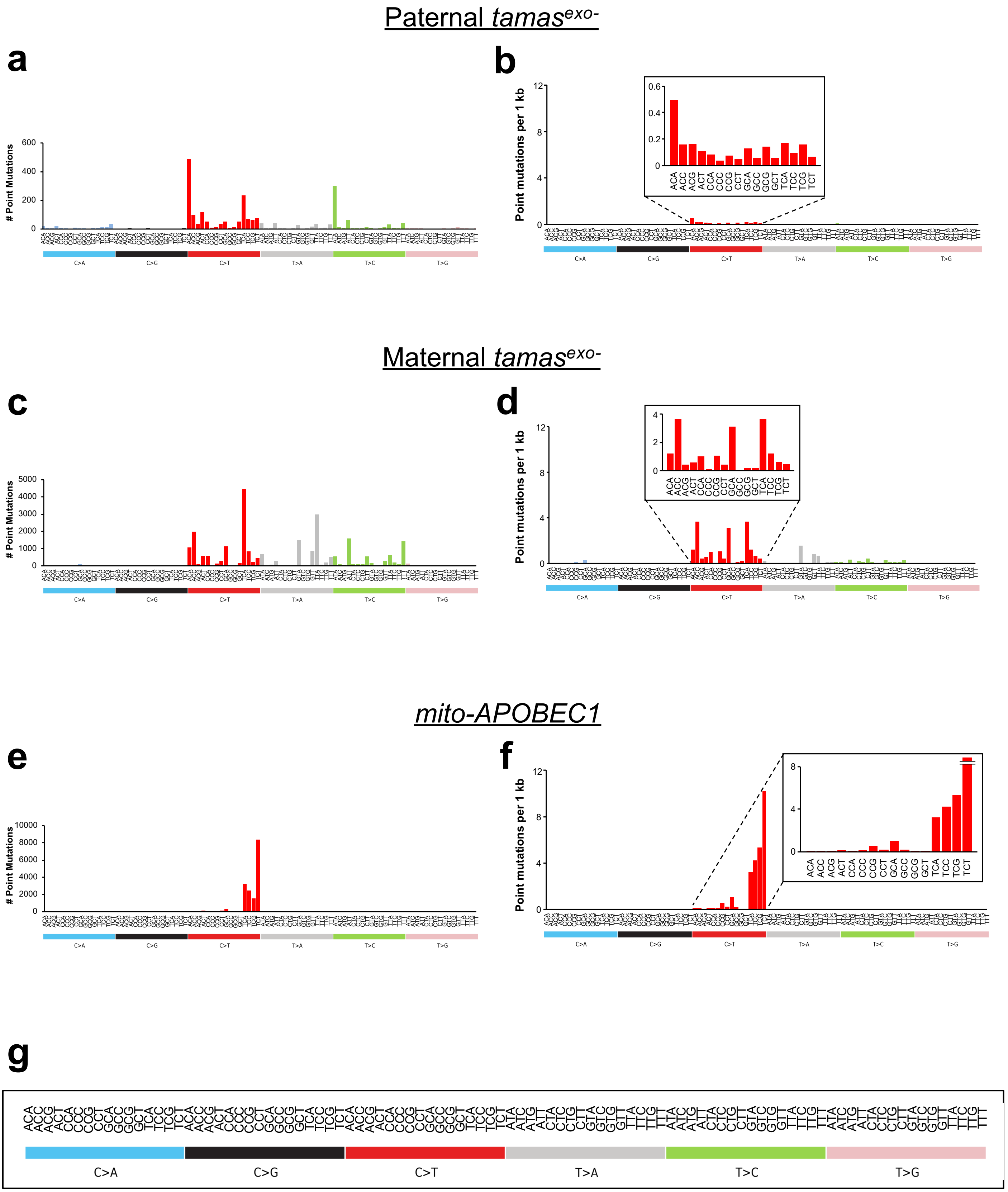

Supplementary Figure 7. *mito-APOBEC1* targets TC dinucleotides with high specificity

(**a**, **c**, **e**) Raw number of C:G and T:A mutations and mutation load (**b**, **d**, **f**) within all 96 possible trinucleotide contexts, detected in 10-day-old flies from (**a**, **b**) paternally inherited *tam*$^{exo-}$ (+/*tam*$^{exo-}$), (**c**, **d**) maternally inherited *tam*$^{exo-}$ (*tam*$^{exo-}$/+) and (**e**, **f**) *mito-APOBEC1* mutator flies. (**g**) An expanded view of all 96 possible trinucleotide contexts, centred on the mutated C:G or T:A. Source data are provided as a Source Data file.
