# Supplementary material for: Mitochondrially-targeted APOBEC1 is a potent mtDNA mutator affecting mitochondrial function and organismal fitness in Drosophila
Source: Nat Commun. 2019 Jul 23;10:3280. doi: 10.1038/s41467-019-10857-y (PMC6650417; doi:10.1038/s41467-019-10857-y)
Supplement: Supplementary file 3 — Description of Additional Supplementary Files [file 41467_2019_10857_MOESM3_ESM.pdf]

Description of Additional Supplementary Files

File name: Supplementary Data 1

Description: Sequence data for point mutations in 10-day-old flies. Tables summarising the point mutation loads for different heteroplasmies for all genotypes analysed at 10 days of age, and the full details for individual animals sequenced. Genotype and animal are indicated on each sheet. Position, nucleotide position in the *Drosophila* reference genome; Reference, annotated nucleotide at the corresponding position; Encoding, gene type: protein coding, tRNA, rRNA, D-loop (A/T-rich region), non-coding (NC); Description, gene name; DCS, duplex consensus sequence, i.e. number of reads at the corresponding position; NT change, nucleotide change (where mutations); % Heteroplasmy, mutation frequency at the defined position; MutPred score, MutPred score at protein-coding genes. Mutations in tRNA and rRNA, that cannot be analysed using the MutPred software, are denoted by ".".

File name: Supplementary Data 2

Description: Sequence data for insertions and deletions in 10-day-old flies. Tables summarising the insertion and deletion loads for all genotypes analysed at 10 days of age, and the full details for individual animals sequenced. Genotype and animal are indicated on each sheet. Position, nucleotide position in the *Drosophila* reference genome; Reference, annotated nucleotide at the corresponding position; Encoding, gene type: protein coding, tRNA, rRNA, D-loop (A/T-rich region), non-coding (NC); Description, gene name; DCS, duplex consensus sequence, i.e. number of reads at the corresponding position; Inserted NT, description of inserted nucleotides (when applicable); Deleted NT, description of deleted nucleotides (when applicable); % Heteroplasmy, frequency of insertion/deletion occurrence(s) at the defined position.

File name: Supplementary Data 3

Description: Mutation sequencing data in 2-day-old flies. Tables summarising the point mutation loads for different heteroplasmies for all genotypes tested at 2 days of age.
